# Dynamics of iron metabolism in patients with bloodstream infections: a time-course clinical study

**DOI:** 10.1038/s41598-023-46383-7

**Published:** 2023-11-06

**Authors:** Hiroshi Moro, Yuuki Bamba, Kei Nagano, Mariko Hakamata, Hideyuki Ogata, Satoshi Shibata, Hiromi Cho, Nobumasa Aoki, Mizuho Sato, Yasuyoshi Ohshima, Satoshi Watanabe, Toshiyuki Koya, Toshinori Takada, Toshiaki Kikuchi

**Affiliations:** Department of Respiratory Medicine and Infectious Diseases, Niigata University Graduate School of Medical and Dental Sciences, 1-757 Asahimachi-Dori, Chuo-Ku, Niigata, 951-8510 Japan

**Keywords:** Clinical microbiology, Infectious-disease diagnostics, Biomarkers, Infection, Acute inflammation, Sepsis, Infectious diseases, Iron

## Abstract

The close relationship between infectious diseases and iron metabolism is well known, but a more detailed understanding based on current knowledge may provide new insights into the diagnosis and treatment of infectious diseases, considering the growing threat of antibiotic-resistant bacteria. This study investigated adult patients with bloodstream infections, temporal changes, and relationships between blood levels of iron and related markers, including hepcidin and lipocalin-2 (LCN2). We included 144 samples from 48 patients (mean age 72 years, 50% male), with 30 diagnosed with sepsis. During the acute phase of infection, blood levels of hepcidin and LCN2 increased rapidly, whereas iron levels decreased, with values in 95.8% of cases below the normal range (40–188 μg/dL). Later, hepcidin and LCN2 decreased significantly during the recovery phase, and the decreased iron concentrations were restored. In the case of persistent inflammation, iron remained decreased. Acute LCN2 levels were significantly higher in patients with sepsis (p < 0.01). Hypoferremia induced by increased hepcidin would reduce iron in the environment of extracellular pathogens, and the increased LCN2 would inhibit siderophores, resulting in the prevention of the pathogen’s iron acquisition in each manner during the acute phase of bloodstream infection.

## Introduction

The link between altered iron balance and infection has been recognized since the mid-twentieth century^1^. Iron is an essential mineral for the human body, playing a critical role in oxygen transport as part of hemoglobin as well as various other physiological functions. However, unbound iron can generate toxic free radicals that damage host tissues^2,3^. Therefore, the iron concentration in the blood is maintained as low as possible and is tightly chaperoned by transferrin. Simultaneously, iron is also an essential nutrient for bacteria, which synthesize and secrete siderophores, small molecules with a high affinity for iron, extracellularly to acquire iron. Therefore, during infection, the host and pathogens compete for iron acquisition^2–4^.

The discovery of hepcidin, a key regulator of iron metabolism, in the early 2000s^5–7^ is considered a major turning point in the field. Hepcidin binds to ferroportin, which is responsible for iron transport and promotes its intracellular degradation^8^. Ferroportin, which is expressed in cells such as small intestinal epithelial cells and macrophages, pumps iron into the blood, and hepcidin regulates the iron utilization cycle in vivo by directly inhibiting ferroportin^9–11^. Hepcidin production is subject to multifaceted regulation by inflammation, body iron levels, tissue injury, and oxygenation status^2^. During inflammation, hepatocytes produce hepcidin via interleukin-6 (IL-6) and signal transducer and activator of transcription 3 (STAT3)-mediated pathways^3,4^.

Another factor involved in iron metabolism is the secreted glycoprotein lipocalin-2 (LCN2). Neutrophil gelatinase-associated lipocalin (NGAL) is another name for LCN2, siderocallin, or 24p3 and belongs to the group of lipophilic small-molecule transporters^12^. Although LCN2 does not bind iron in isolation^13^, it primarily binds to bacterial siderophores, inhibiting their iron uptake and thus limiting bacterial iron uptake^14^. During infection, neutrophils, macrophages, epithelial cells, and other cells produce LCN2. Inflammatory mediators, such as tumor necrosis factor (TNF)-α, IL-1β, and IL-6, cause this reaction^15^, implying its involvement in the innate immune system’s antimicrobial iron restriction strategy.

While factors involved in iron metabolism are becoming increasingly clear, the relationship between iron metabolism and infection is often presented in the context of chronic inflammation and anemia^16,17^. In addition, the current understanding of the relationship between iron metabolism and infection, particularly in acute infection models, is primarily derived from animal studies^18^. Thus, there remains scope for further exploration in understanding iron metabolism in the realm of human infectious diseases. In addition, in the modern context of the emergence of drug-resistant bacteria, a new approach to the pathophysiology of infectious diseases is urgently needed, and iron metabolism is considered a particularly attractive target. Against this background, this research aims to further investigate infectious diseases through the axis of iron metabolism, focusing on hepcidin and LCN2. To this end, we have comprehensively analyzed iron metabolism and inflammatory markers during bloodstream infection using clinical samples.

## Results

Table 1 summarizes the participants’ clinical characteristics. This study included 144 clinical samples from 48 patients (median age 72 years, 50.0% male). Among the blood tests included in this study, there were missing values for neutrophil count (1 case, 3 samples) and ferritin (7 cases, 21 samples). Nineteen (39.6%) cases were hospital-onset, and 29 (60.4%) were community-onset. The median Sequential Organ Failure Assessment (SOFA) score at D1 was 2 points (range 0–14 points), and no deaths were observed. A total of 30 of 48 patients (62.5%) were diagnosed with sepsis. Underlying conditions included urinary tract (25.0%), biliary (20.8%), and catheter-related (14.6%) infections. A total of 55 organisms were detected in blood cultures, with six cases of multiple organisms (Table S1). *Escherichia coli* was the most commonly isolated (27.3%), followed by *Klebsiella pneumoniae* (9.1%), *K. oxytoca* (5.5%), and *P. aeruginosa* (5.5%). Fungi were detected in five cases, all of which were *Candida *spp.

**Table 1:** Characteristics of participants.

Characteristic (n = 48)			
Age, years	Median (IQR)	72	(65–80)
Gender, male	n (%)	24	(50.0%)
Sepsis	n (%)	30	(62.5%)
SOFA score*	Median (IQR)	2	(1–4)
Comorbidity	n (%)		
Diabetes mellitus		17	(35.4%)
Autoimmune disease		12	(25.0%)
Solid organ malignancy		19	(39.6%)
Hematologic malignancy		7	(14.6%)
Chronic heart failure		4	(8.3%)
Chronic renal failure		10	(20.8%)
Chronic respiratory failure		1	(2.1%)

Data are expressed as median (IQR) or n (%).

*SOFA score* Sequential Organ Failure Assessment score.

*Value at D1.

### Iron kinetics and associated markers

**Figure 1:**
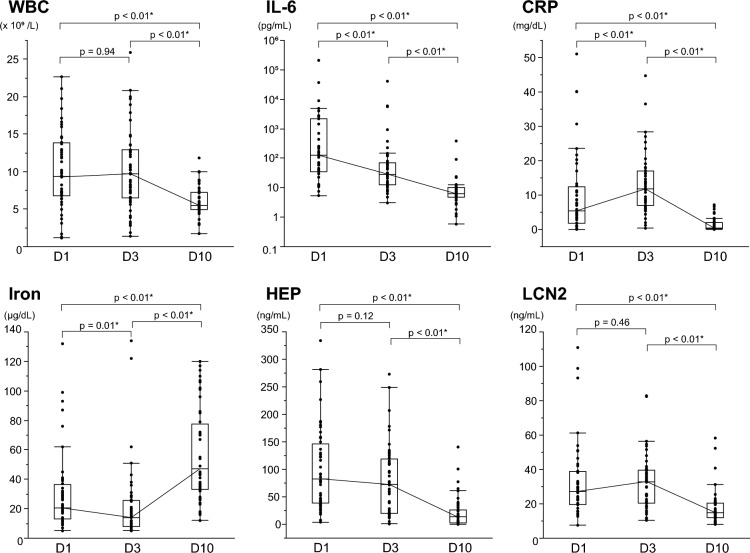
Changes over time in markers of iron metabolism and inflammation in bloodstream infections. Changes over time in inflammatory and iron parameters in patients with bloodstream infections; WBC (**a**), IL-6 (**b**), CRP (**c**), Iron (**d**), HEP (**e**) and LCN2 (**f**) levels were measured at each time point and are shown in box-and-whisker plots. The thick line represents the median, and the boxes represent the interquartile range. The median of each point is connected by a solid line. Logarithmic scale is used for the IL-6 Y-axis. Measurements were compared between points, and P-values were calculated using the Wilcoxon signed-rank test. Statistically significant items are marked with an asterisk (*). *WBC* white blood cell count, *IL-6* interleukin-6, *CRP* C-reactive protein, *HEP* hepcidin, *LCN2* lipocalin-2.

During the clinical course of the bloodstream infections, the temporal changes of the iron metabolism and inflammation markers are shown in Fig. 1. In the context of this study, the values measured at D10 were considered the baseline against which the values measured at D1 and D3 were compared. Blood levels of inflammatory markers such as interleukin 6 (IL-6) and C-reactive protein (CRP), as well as white blood cell (WBC) count, rose during the infection's acute period (D1 and D3). Additionally, blood levels of iron-related hepcidin and LCN2 also increased when compared to D10. In contrast, iron levels decreased, with 95.8% of cases having iron levels below the normal range (40–188 μg/dL) at D1 or D3. In the recovery phase (D10), as the immune system and treatment brought the infection under control, WBC and levels of IL-6, CRP, hepcidin, and LCN2 substantially decreased compared to the acute phase of infection. Iron levels showed a recovery trend and increased to within the reference range in 62.5% of cases. During the study, the median change in iron concentration was 31 μg/dL (maximum 104 μg/dL, IQR 21–63 μg/dL). Table 2 presents representative values for all measured parameters at each point. Another marker related to iron metabolism is ferritin, increased during the acute phase and then decreased like other acute-phase proteins. In contrast, unsaturated iron-binding capacity (UIBC) and total iron-binding capacity (TIBC), like iron, decreased during the acute phase of infection. TIBC increased significantly during the recovery phase, whereas UIBC did not.

**Table 2:** Laboratory results at each time point.

Analyte (units)	Measured value			p value		
	D1	D3	D10	D3-D1	D10-D3	D10-D1
WBC (× 10^9/L)	9.4 (6.8–13.9)	9.7 (6.5–12.9)	5.5 (5.0–7.2)	0.94	< 0.01**	< 0.01*
NEU (× 10^9/L)	7.0 (5.5–12.7)	9.1 (5.0–11.6)	3.6 (2.5–4.7)	0.90	< 0.01*	< 0.01*
Hb (g/L)	112.8 ± 2.6	103.7 ± 2.2	104.5 ± 2.6	< 0.01*	0.82	0.02
PLT (× 10^9/L)	173 (135–218)	149 (115–220)	251 (207–328)	0.19	< 0.01*	< 0.01*
ALB (g/L)	30.5 ± 1.0	26.0 ± 0.9	30.4 ± 1.0	0.01*	< 0.01*	1.00
CRE (mg/dL)	1.0 (0.7–1.5)	1.1 (0.7–1.6)	0.7 (0.6–1.0)	0.76	< 0.01*	< 0.01*
TBIL (mg/dL)	0.9 (0.5–1.4)	0.8 (0.4–1.3)	0.5 (0.4–0.8)	0.36	< 0.01*	< 0.01*
IL-6 (pg/mL)	130.1 (35.3–2189.7)	28.2 (12.6–70.0)	6.3 (4.7–10.4)	< 0.01*	< 0.01*	< 0.01*
CRP (mg/dL)	5.4 (1.9–12.3)	11.8 (7.0–16.9)	0.4 (0.1–1.9)	< 0.01*	< 0.01*	< 0.01*
P-SEP (pg/mL)	697 (364–1063)	1075 (459–1920)	266 (183–449)	0.07	< 0.01*	< 0.01*
Iron (µg/dL)	21 (13–36)	14 (8–26)	47 (33–77)	0.01*	< 0.01*	< 0.01*
UIBC (µg/dL)	223 ± 9	187 ± 9	208 ± 11	0.01*	0.14	0.26
TIBC (µg/dL)	253 ± 9	209 ± 9	265 ± 11	< 0.01*	< 0.01*	0.38
TSAT (%)	8.1 (5.1–13.5)	7.5 (4.4–12.2)	19.0 (13.4–30.3)	0.25	< 0.01*	< 0.01*
FER (ng/mL)	171 (69–341)	201 (106–416)	111 (53–204)	0.24	< 0.01*	0.14
HEP (ng/mL)	83.1 (38.5–146.6)	72.3 (20.4–119.3)	13.7 (2.7–26.2)	0.12	< 0.01*	< 0.01*
LCN2 (ng/mL)	27.1 (19.6–39.0)	32.7 (20.4–39.5)	14.6 (12.1–20.5)	0.46	< 0.01*	< 0.01*

Data are expressed as median (interquartile range) or mean ± standard error. Asterisks denote significant differences (p < 0.05).

*WBC* white blood cell count, *NEU* neutrophil count, *Hb* hemoglobin, *PLT* platelet count, *ALB* albumin, *CRE* creatinine, *TBIL* total bilirubin, *IL-6* interleukin 6, *CRP* C-reactive protein, *P-SEP* presepsin, *TIBC* total iron-binding capacity, *UIBC* unsaturated iron-binding capacity, *TSAT* transferrin saturation, *FER* ferritin, *HEP* hepcidin, *LCN2* lipocalin-2.

Furthermore, a line graph (Fig. S1) shows case-specific iron and inflammatory markers trends in the 19 hospital-onset cases. The community-onset cases were excluded to make the time from onset to positive blood culture (D1) as uniform as possible. Measurements at each point were compared as related pairs. As a result, a typical variation pattern appeared for each parameter, consistent with the result described above. Among them, CRP and LCN2 were the highest at D3 in most cases (89.5% and 78.9%, respectively), and iron was the lowest at D3 in 89.5% of cases. Hepcidin, on the other hand, was the highest at D1 in most cases (68.4%), indicating earlier hepcidin elevation during the acute phase.

### Comparison based on disease severity and clinical course

**Figure 2:**
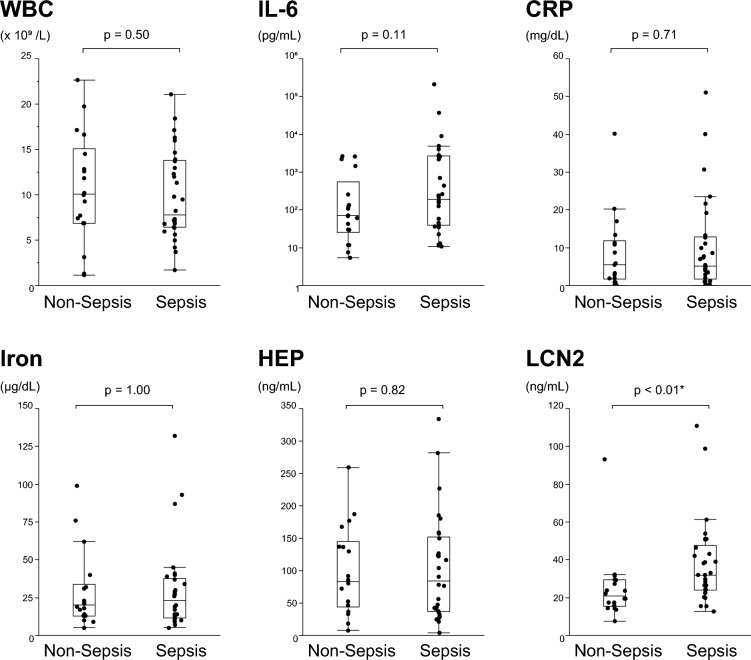
Comparison of laboratory data with or without sepsis. All the participants were divided into two groups: sepsis (n = 36) and non-sepsis (12) groups. WBC (**a**), IL-6 (**b**), CRP (**c**), iron (**d**), HEP (**e**), and LCN2 (**f**) blood levels at D1 were measured and compared between two groups. In box-and-whisker plots, the thick line represents the median, and the boxes represent the interquartile range. The median of each point is connected by a solid line. A logarithmic scale is used for the IL-6 Y-axis. Measurements were compared between points, and P-values were calculated using the Wilcoxon signed-rank test. Statistically significant items are marked with an asterisk (*). *WBC* white blood cell count, *IL-6* interleukin-6, *CRP* C-reactive protein, *HEP* hepcidin, *LCN2* lipocalin-2.

As a measure of severity, blood test results were compared between groups at points D1 and D3, the acute phase, and participants were split into two groups based on the presence or absence of sepsis (Fig. 2, Table S2). Among the iron metabolism indicators, LCN2 was significantly higher in sepsis at D1 and D3 (p < 0.01 and p = 0.03, respectively). With or without sepsis, laboratory studies on D10 revealed no significant differences.

We also examined correlations between the SOFA score and various measured parameters at each time point to assess the temporal progression of disease severity (Table 3). Excluding parameters intrinsic to the SOFA score, such as platelet count, creatinine, and total bilirubin, significant correlations with the SOFA score were observed for presepsin, IL-6, and LCN2 at D1, with presepsin also showing a correlation at D3. Markers related to iron metabolism other than LCN2, including hepcidin, showed no clear correlation with SOFA scores.

**Table 3:** Correlations of laboratory parameters with SOFA score over time.

	SOFA D1	SOFA D3	SOFA D10
WBC	− 0.02	0.10	− 0.05
NEU	0.01	0.19	0.06
Hb	− 0.06	− 0.16	− 0.20
PLT	− 0.49*	− 0.67**	− 0.29
ALB	− 0.08	− 0.18	− 0.15
CRE	0.71**	0.56**	0.39*
TBIL	0.40*	0.43*	0.15
IL-6	0.43*	0.24	− 0.04
CRP	0.12	0.24	0.25
P-SEP	0.50**	0.42*	0.30*
Iron	− 0.13	− 0.26	0.27
UIBC	− 0.10	− 0.12	− 0.12
TIBC	− 0.05	− 0.15	− 0.11
TSAT	− 0.15	− 0.19	0.27
FER	− 0.04	− 0.10	0.11
HEP	0.05	0.14	0.06
LCN2	0.36*	0.15	0.25

Correlation coefficients with absolute values between 0.3 and 0.5 are marked * and those greater than 0.5 are marked **.

*WBC* white blood cell count, *NEU* neutrophil count, *Hb* hemoglobin, *PLT* platelet count, *ALB* albumin, *CRE* creatinine, *TBIL* total bilirubin, *CRP* C-reactive protein, *P-SEP* presepsin, *IL-6* interleukin 6, *TIBC* total iron-binding capacity, *UIBC* unsaturated iron-binding capacity, *TSAT* transferrin saturation, *FER* ferritin, *HEP* hepcidin, *LCN2* lipocalin-2.

Additionally, we evaluate the dynamics of iron markers during persistent inflammation. Based on previous reports proposing criteria for persistent inflammation, immunosuppression, and catabolism syndrome^19^, patients with CRP levels exceeding 2.0 mg/dL at D10 (median 0.36 mg/dL) were defined as the persistent inflammation group (n = 11) and the other patients as the control group (n = 37). The two groups’ laboratory values at D10 were then compared (Table 4). Notably, iron levels were significantly lower in the persistent inflammation group (33 µg/dL; IQR 26–44) compared to the control group (67 µg/dL; IQR 39–91; p < 0.01). Furthermore, hemoglobin, albumin, and total bilirubin were significantly lower in the persistent inflammation group, while IL-6, presepsin, and LCN2 were significantly higher compared to the control group.

**Table 4:** Comparison of laboratory data with or without persistent inflammation.

Analyte (units)	Control	Persistent inflammation^a^	p value
	(n = 37)	(n = 11)	
WBC (× 10^9/L)	5.4 (4.9–7.2)	5.7 (5.3–8.9)	0.45
NEU (× 10^9/L)	3.4 (2.5–4.6)	4.3 (3.5–7.8)	0.09
Hb (g/L)	108.6 ± 2.9	90.8 ± 3.5	< 0.01*
PLT (× 10^9/L)	239 (203–324)	257 (217–342)	0.70
ALB (g/L)	31.6 ± 1.2	26.0 ± 1.6	0.02*
CRE (mg/dL)	0.7 (0.5–1.0)	0.9 (0.7–1.1)	0.07
TBIL (mg/dL)	0.6 (0.4–0.9)	0.4 (0.3–0.5)	0.04*
IL-6 (pg/mL)	5.8 (3.8–9.6)	9.3 (5.9–22.4)	0.02*
P-SEP (pg/mL)	242 (168–440)	336 (250–486)	0.048*
Iron (µg/dL)	67 (39–91)	33 (26–44)	< 0.01*
UIBC (µg/dL)	216 ± 13	178 ± 19	0.10
TIBC (µg/dL)	281 ± 11	210 ± 19	< 0.01*
TSAT (%)	22.4 (13.9–32.7)	15.5 (9.4–20.3)	0.12
FER (ng/mL)	118 (49–250)	106 (58–178)	0.82
HEP (ng/mL)	11.8 (1.9–24.3)	19.3 (3.2–33.6)	0.15
LCN2 (ng/mL)	14.1 (10.5–19.5)	16.2 (15.3–25.5)	0.02*

*WBC* white blood cell count, *NEU* neutrophil count, *Hb* hemoglobin, *PLT* platelet count, *ALB* albumin, *CRE* creatinine, *TBIL* total bilirubin, *IL-6* interleukin 6, *CRP* C-reactive protein, *P-SEP* presepsin, *TIBC* total iron-binding capacity, *UIBC* unsaturated iron-binding capacity, *TSAT* transferrin saturation, *FER* ferritin, *HEP* hepcidin, *LCN2* lipocalin-2.

Data are expressed as median (interquartile range) or mean ± standard error. Asterisks denote significant differences (p < 0.05). P-values near the significance boundary are represented with three decimal places for precision.

^a^Persistent inflammation is defined in this study following the criteria outlined in Nakamura et al.^19^.

### Correlations between items measured

**Figure 3:**
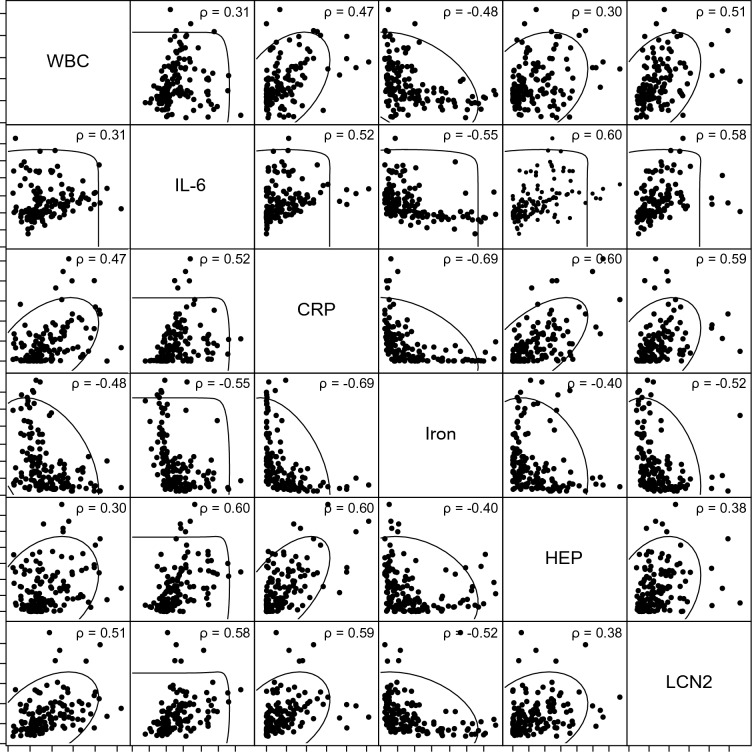
Correlations between iron and inflammatory markers. Scatterplot matrices are shown for white blood cell count, inflammatory markers, and iron metabolism markers. Probability ellipses (α = 0.95) and Spearman's rank correlation coefficient (ρ) were described. The logarithmic scale is used for the IL-6 axis. Table S2 lists additional variables. *WBC* white blood cell count, *IL-6* interleukin-6, *CRP* C-reactive protein, *HEP* hepcidin, *LCN2* lipocalin-2.

Correlations between analytes were evaluated for 144 samples from D1 to D10 (Fig. 3). Tests that showed moderate negative correlations with iron concentration were CRP (ρ =  − 0.69), neutrophils (ρ =  − 0.55), IL-6 (ρ =  − 0.55), and LCN2 (ρ =  − 0.52), and those with weak negative correlations were WBC count (ρ =  − 0.48), presepsin (ρ =  − 0.45) and hepcidin (ρ =  − 0.40). On the other hand, the iron concentration showed a weak positive correlation with albumin and TIBC (both ρ = 0.39). Hepcidin showed a moderate positive correlation with CRP (ρ = 0.60), IL-6 (ρ = 0.60), and ferritin (ρ = 0.52). LCN2 showed a moderate positive correlation with CRP (ρ = 0.59), neutrophils (ρ = 0.59), IL-6 (ρ = 0.58), and WBC (ρ = 0.51). Table S3 shows the correlation coefficients between all measured parameters (17 items).

**Figure 4:**
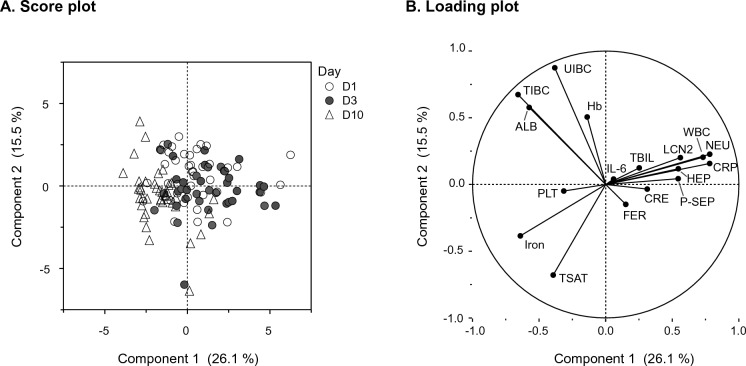
Principal component analysis for all measured items. Principal component analysis identified the first two principal components, which explained 41.6% of the variation in the dataset. (**A**) Score plot showing each principal component’s score for each sample. Circles, filled circles, and triangles indicate D1, D3 and D10, respectively. (**B**) Loading plot showing the items loading on each principal component. Items clustered close together on the graph indicate that they vary in the same manner. *WBC* white blood cell count, *NEU* neutrophil count, *Hb* hemoglobin, *PLT* platelet count, *ALB* albumin, *CRE* creatinine, *TBIL* total bilirubin, *IL-6* interleukin-6, *CRP* C-reactive protein, *P-SEP* presepsin, *TIBC* total iron-binding capacity, *UIBC* unsaturated iron-binding capacity, *TSAT* transferrin saturation, *FER* ferritin, *HEP* hepcidin, *LCN2* lipocalin-2.

Principal component analysis (PCA) was performed on all samples measured from D1 to D10. Table S4 displays the eigenvectors of principal components 1 (PC1) to 4 (PC4). The first two PCs accounted for 41.6% of the variation in the data set. For PC1, variables related to infection and inflammation such as WBC, neutrophils, and presepsin predominated with high positive eigenvector values. For PC2, albumin and transferrin (represented by TIBC) predominated with large positive eigenvector values. Score plots revealed the data distribution without outliers (Fig. 4A). While D1 and D3 samples appeared to have similar clustering patterns, D10 samples revealed a distinct distribution. This shift from D1 and D3 to D10 indicates dynamic changes in the influence of the parameters over time. In the loading plot (Fig. 4B), both hepcidin and LCN2 were found to cluster with WBC, neutrophils, presepsin, and CRP. Conversely, iron clustered in the opposite quadrant to these parameters, suggesting a negative correlation.

## Discussion

Our study comprehensively evaluated host iron metabolism in bloodstream infections using clinical blood samples. We detailed the temporal dynamics of several parameters related to iron metabolism and inflammatory markers, focusing on the dynamics of hepcidin, the master regulator of iron. A rapid increase in the acute phase typically characterized the time course of blood hepcidin levels. This outcome was consistent with a prior experiment using an lipopolysaccharide (LPS) injection^20^ and a typhoid infection^21^ on a human volunteer. In contrast to hepcidin, iron levels decreased rapidly, although the iron levels tended to decrease later than the increase in hepcidin levels during the acute phase. Because the present study targeted bloodstream infections, the effect of hepcidin may contribute to the host’s innate immunity by reducing the amount of iron in the extracellular pathogen’s environment, thereby inhibiting iron acquisition. This observation is consistent with previous findings^2,3,18^.

In contrast, iron is an essential nutrient for host homeostasis, and persistent iron deficiency in the blood should be avoided^2^. In this study, hypoferremia in the acute phase was transient. As the infection was controlled and resolved, there was a progressive decrease in hepcidin levels and a corresponding increase in iron levels. If inflammation persisted, iron levels remained low for a prolonged period. Our time course analysis provides a clearer and more comprehensive understanding of how the human body manages and regulates iron during these critical periods.

The current study also includes blood levels of another iron-related factor, LCN2. It is engaged in several physiological and pathophysiological processes, such as inflammation, infection, immune response, and metabolic homeostasis^22^. The present results show that blood levels of LCN2 increase during the acute phase of systemic infection and decrease during the recovery phase, consistent with a previous report using a mouse model^23^. Furthermore, LCN2 levels were significantly elevated during the acute phase of sepsis and showed a significant correlation with the D1 SOFA score, indicating its potential as a marker of sepsis severity. The LCN2 knockout mice also showed a protective role in sepsis, as organ damage and mortality were worse in LCN2 knockout mice than in wild-type mice after LPS injection^23^. Therefore, the present results suggest an important action of LCN2 in sepsis pathogenesis, warranting further study. LCN2 is considered an effective biomarker of acute kidney injury^24^. In addition to a strong inflammatory response, LCN2 levels may have been affected by the kidney injury associated with bloodstream infection, including sepsis, in the present study. However, no correlation was found between creatinine and LCN2 levels, and the effect was considered limited in the study.

The present study reiterated the close relationship between inflammation and iron metabolism in acute systemic infections. A possible starting point for these findings is the involvement of the inflammatory cytokine IL-6, released by immune cells in response to infection or injury. In a previous report, plasma iron and TIBC levels decreased and then recovered after IL-6 administration in animal models^25^. In general, blood levels of IL-6 increase rapidly during the early stages of infection, from hours to days^27^. Acute-phase proteins like CRP, ferritin, hepcidin, and LCN2 are induced by IL-6^3,4,15,26^. On the other hand, IL-6 decreases albumin and transferrin production^27^. These effects of IL-6 were consistent with the results of the current study, including time trends of each measure, correlations, and PCA.

In recent years, the spread of multidrug-resistant bacteria has become a global problem, and the development of antibiotics with new mechanisms of action is urgently needed. In this setting, the bacterial iron transport mechanism is an attractive site of action for antimicrobial agents. Siderophore cephalosporins can efficiently penetrate the outer membrane through the bacterial iron transport system by forming iron chelate complexes^28^ and are reportedly effective against multidrug-resistant Gram-negative bacteria^29–31^. Therefore, the dynamics of iron metabolism in the clinical course of this study are expected to be useful for the effective use of such antimicrobial agents. Under iron-deficient conditions, bacteria upregulate the siderophore and the iron transporter system^32,33^. Consistent with this finding, in vitro studies have shown that the concentration of iron in the culture medium affects the antimicrobial activity of siderophore cephalosporins, with lower minimum inhibitory concentrations in iron-deficient situations^28^. Therefore, the hypoferremia shown in this study during the acute phase of systemic infections may also enhance the efficacy of such antimicrobial agents in vivo.

There are several limitations to this research. First, it was a single-center study conducted at a university hospital and included a relatively small number of patients with diverse clinical backgrounds, and the possibility of unintentional selection bias cannot be excluded. Second, it was not possible to establish baseline values for each parameter before disease onset. It was difficult to determine the onset of bloodstream infections in advance, so the values measured during the recovery phase, D10, were used for comparison. Third, because there were no deaths in this study, we could not examine the relationship between poor prognoses and iron metabolism in bloodstream infections. To address this issue, a sepsis group and a persistent inflammation group were established to investigate differences in severity and pathophysiology. Nevertheless, despite these limitations, we believe that the study sheds light on the intricate mechanisms of iron metabolism during systemic infection and provides a foundation for future studies.

In conclusion, it was suggested that hypoferremia due to the effect of hepcidin decreases iron in the environment of extracellular pathogens, the increase in LCN2 levels directly inhibits siderophores during the acute phase of infection, and these effects of hepcidin and LCN2 associated with the inflammatory response prevents iron acquisition by the pathogen in each manner. In an era of prevalent drug-resistant bacteria, a detailed understanding of the infection–iron axis in clinical practice is critical and represents a potential advance in the diagnosis and treatment of infectious diseases in the future.

## Methods

### Ethics approval and consent to participate

This study was conducted in compliance with the Declaration of Helsinki and current ethical guidelines. The Ethics Committee of Niigata University (Approval Number: 2015-2301) approved the study, including the waiver of written informed consent due to the use of residual blood samples in the study and the absence of novel invasive procedures for patients. Information about the study’s goals and an opt-out option were provided on the official website of Niigata University School of Medicine.

### Study participants and design

At the Niigata University Medical and Dental Hospital (827 beds, tertiary urban hospital), the study included cases of bloodstream infections. The study period was from March 2015 to December 2016, and patients with new positive blood cultures were reviewed daily. A positive blood culture confirmed the diagnosis of bloodstream infection, and single positive cultures of normal skin flora were excluded.

During each case’s clinical course, the day of the first positive blood culture was defined as day 1 of illness (D1). Previous in vivo reports indicated that hepcidin reaches its maximum concentration in about 6 h^21^ and C-reactive protein (CRP) in 2–3 days^34^. Based on these findings, this study included the test results of each item at two points, D1 and days 2 to 3 of illness (D3), as the acute phase of infection. Additionally, the first day of blood testing after day 10 of illness was defined as D10 and treated as an indicator in the recovery phase. For the purpose of this study, the baseline values of each parameter were defined based on the measurements taken at D10. Eligible patients were adults aged ≥ 18 years who had a blood test at the appropriate time and had residual blood samples available. Treating physicians obtained the laboratory results used in this study during their practice. For missing data and additional testing, the residual plasma samples of D1, D3, and D10 stored at − 80°C were used; no additional blood samples were collected for this study. Finally, 150 samples and 50 cases were obtained, but 6 samples and 2 cases were excluded because there were not enough samples for additional testing.

### Data collection

Clinical information, such as age, gender, medical history, medications, and the microorganisms that caused the infection, was gathered from computerized medical records. To assess the number and severity of organ failures in the cases, the SOFA scores^35^ were obtained each time. Sepsis diagnoses were based on international criteria^36^. Laboratory test results included WBC count (reference range: 3.3–8.6 cells × 10^9/L), neutrophil count (1.6–6.0 cells × 10^9/L), hemoglobin (male: 137–168, female: 116–148 g/L), platelet count (158–348 cells × 10^9/L), albumin (41–51 g/L), creatinine (male: 0.65–1.07, female 0.46–0.79 mg/dL), total bilirubin (0.4–1.5 mg/dL), and CRP (< 0.15 mg/dL). Additionally, residual or stored plasma was used to measure IL-6 (≤ 7 pg/mL), presepsin (< 500 pg/mL), iron (40–188 µg/dL), TIBC (250–460 µg/dL), UIBC (191–269 µg/dL), ferritin (male: 13–277, female 5–152 ng/mL), transferrin saturation (TSAT, 20–50%), hepcidin, and LCN2. Using a quick chemiluminescent enzyme immunoassay, plasma presepsin was determined (PATHFAST Immunoanalyzer; LSI Medience Corporation, Tokyo, Japan). TIBC measurement substituted for transferrin. TSAT was based on the ratio of iron to TIBC (TSAT = iron ÷ TIBC × 100). Hepcidin has several isoforms^37^, and 25 amino acid of hepcidin-25, the active form of hepcidin, were included in this study. As previously reported, surface-enhanced laser desorption/ionization time-of-flight mass spectrometry (SELDI-TOF MS) was used to measure plasma hepcidin levels^38^. Although the reference values of hepcidin were not determined, the median value of 17 healthy volunteers was measured to be 19.1 ng/mL using the same method^39^. An enzyme-linked immunosorbent assay kit (Hycult Biotech, Pennsylvania, USA) determined LCN2 plasma levels with internal controls according to the kit instructions.

### Statistical analysis

JMP 14.2 (SAS Institute Inc., NC, USA) was used to analyze all data. The Shapiro–Wilk test was employed to ascertain whether continuous factors had a normal distribution. Continuous variables were summarized by mean ± standard error in the case of a normal distribution and by the median and interquartile range (IQR) in other cases. When the distribution was normal, the student’s t-test was applied for evaluations between the two groups. The two-tailed F-test determined equivariance, and Welch’s t-test was used when the two groups were unequally distributed. The Wilcoxon signed-rank test was applied when the distribution was non-normal, and Wilcoxon's signed-rank sum test was applied to analyze paired data with a non-normal distribution. Statistical significance was defined as p < 0.05.

The strength and direction of the linear connection between the two variables were evaluated using correlation coefficients. Pearson's correlation coefficient was used when both factors were normally distributed; otherwise, Spearman's correlation value was applied. For missing values, correlations were estimated using the pairwise method. Correlation coefficients with absolute values between 0.3 and 0.5 were considered a weak correlation, those between 0.5 and 0.7 had a moderate correlation, and those above 0.7 had a high correlation.

The multidimensional data structure was represented succinctly using PCA, which was also used to visualize the data's characteristics and show connections between the variables. It constructs principal components, which are eigenvectors, of the covariance matrix of the data, representing directions in the data that explain a maximum amount of variance^40^. The Scores plot graphs the calculated values of each component in relation to each other, with each value adjusted for mean and standard deviation. The loading plot graphs the unrotated loading matrix between the variables and the components. Values close to 1 indicate a greater effect of this component^41^.

### Supplementary Information

Supplementary Information 1.

Supplementary Information 2.

## Data Availability

All data analyzed during this study are included in Supplementary Information File 2.
