# Supplementary material for: Dynamics of iron metabolism in patients with bloodstream infections: a time-course clinical study
Source: Sci Rep. 2023 Nov 6;13:19143. doi: 10.1038/s41598-023-46383-7 (PMC10628148; doi:10.1038/s41598-023-46383-7)
Supplement: Supplementary file 1 — Supplementary Information 1. [file 41598_2023_46383_MOESM1_ESM.pdf]

Supplemental Material

Dynamics of Iron Metabolism in Patients with Bloodstream Infections: A Time-Course Clinical Study

Hiroshi Moro[#], Yuuki Bamba[#], Kei Nagano, Mariko Hakamata, Hideyuki Ogata, Satoshi Shibata, Hiromi Cho, Nobumasa Aoki, Mizuho Sato, Yasuyoshi Ohshima, Satoshi Watanabe, Toshiyuki Koya, Toshinori Takada, and Toshiaki Kikuchi

Department of Respiratory Medicine and Infectious Diseases, Niigata University Graduate School of Medical and Dental Sciences, 1-757 Asahimachi-dori, Chuo-ku, Niigata 951-8510, Japan

[#]Contributed equally.

Correspondence

Hiroshi Moro

Department of Respiratory Medicine and Infectious Diseases Niigata University Graduate School of Medical and Dental Sciences, 1-757 Asahimachi-dori, Chuo-ku, Niigata 951-8510, Japan,

Tel: +81-25-368-9325, Fax: +81-25-368-9326,

E–mail: hmoro@med.niigata-u.ac.jp

Figure Legends

Figure S1

Case-specific trends in iron and inflammatory markers over time

Note. Case-specific time course of iron metabolism and inflammatory parameters in patients with hospital-onset bloodstream infections (n = 19). Each connected line represents a single patient. The logarithmic scale is used for the IL-6 Y-axis. WBC (a), IL-6 (b), CRP (c), Iron (d), HEP (e), LCN2 2 (f).

Abbreviations. WBC, white blood cell count; IL-6, interleukin-6; CRP, C-reactive protein; HEP, hepcidin; LCN2, lipocalin-2.

Figure S1 *Case-specific trends in iron and inflammatory markers over time*

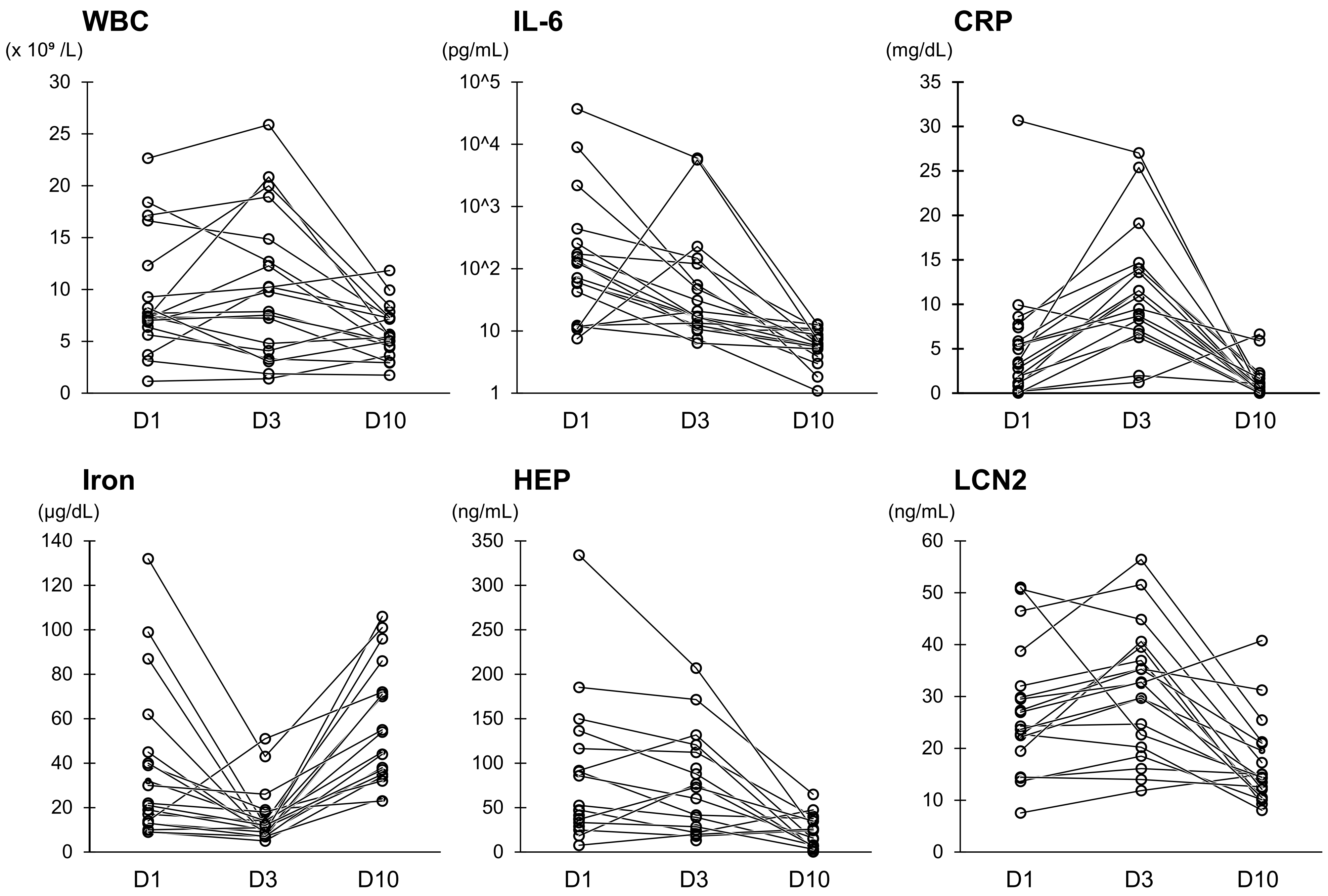

Tables

Table S1

Isolated causative pathogens

**Pathogens (n = 55)**	**n (%)**
Escherichia coli	15 (27.3%)
Klebsiella pneumoniae	5 (9.1%)
Klebsiella oxytoca	3 (5.5%)
Pseudomonas aeruginosa	3 (5.5%)
Aeromonas caviae	2 (3.6%)
Candida parapsilosis	2 (3.6%)
Enterobacter cloacae	2 (3.6%)
Enterococcus faecalis	2 (3.6%)
Haemophilus influenzae	2 (3.6%)
Staphylococcus epidermidis	2 (3.6%)
Streptococcus pyogenes	2 (3.6%)
Miscellaneous	15 (27.3%)

Note. Multiple detections in the same case are included.

Table S2

Comparison of laboratory data with or without sepsis

	D1			D3		
Analyte (Units)	Non-Sepsis [a]	Sepsis [b]	p value	Non-Sepsis [a]	Sepsis [b]	p value
WBC (X 109/L)	10.1 (6.9–15.0)	7.8 (6.4–13.8)	0.50	8.4 (5.4–13.1)	10.4 (7.0–13.0)	0.46
NEU (X 109/L)	9.1 (4.8–12.8)	6.9 (5.8–12.8)	0.85	7.1 (4.1–12.0)	9.5 (5.8–12.0)	0.14
Hb (g/L)	108.5 ± 19.1	115.4 ± 16.6	0.20	101.1 ± 3.6	105.3 ± 2.8	0.35
PLT (X 109/L)	193 (167– 291)	156 (115–194)	0.03*	203 (131–242)	136 (103–169)	<0.01*
ALB (g/dL)	29.1 ± 1.7	31.3± 1.2	0.29	25.4 ± 1.5	26.4 ± 1.1	0.63
CRE (mg/dL)	0.8 (0.6–1.4)	1.2 (0.8–1.6)	0.07	0.9 (0.6–1.3)	1.1 (0.9–1.7)	0.07
TBIL (mg/dL)	0.6 (0.5–1.1)	1.1 (0.9–1.4)	0.02*	0.5 (0.4–0.8)	1.1 (0.7–1.7)	<0.01*
IL-6 (pg/mL)	70.0 (25.2–554.0)	194.1 (38.7–2630.9)	0.11	16.7 (7.8–34.2)	46.3 (14.5–121.8)	0.02*
CRP (mg/dL)	5.5 (1.7–11.8)	5.2 (1.8–12.8)	0.71	11.5 (6.9–15.3)	12.3 (6.8–19.5)	0.66
P-SEP (pg/mL)	513 (375–773)	802 (358–1518)	0.30	609 (442–1201)	1456 (486–2001)	0.15
Iron (µg/dL)	20 (13–34)	23 (12–38)	1.00	15 (10–4)	11 (7–25)	0.13
UIBC (µg/dL)	206 ± 15	233 ± 11	0.15	167 ± 15	199 ± 11	0.09
TIBC (µg/dL)	235 ± 15	263 ± 12	0.15	198 ± 15	216 ± 11	0.33
TSAT (%)	8.4 (5.1–17.6)	7.9 (5.0–12.4)	0.60	8.7 (4.8–15.8)	5.5 (4.1–10.9)	0.12
FER (ng/mL)	191 (71–332)	158 (69–364)	0.84	219 (122–504)	201 (90–393)	0.48
HEP (ng/mL)	83.1 (44.1–144.7)	84.4 (37.1–151.7)	0.82	67.9 (26.3–114.0)	77.1 (19.3–121.6)	1.00
LCN2 (ng/mL)	20.8 (15.4–29.3)	32 (23.9–47.5)	<0.01*	25.6 (17.2–34.2)	35.4 (24.7–42.5)	0.03*

Note. [a] n = 18. [b] n = 30. Data are expressed as median (interquartile range) or mean ± standard error. Asterisks denote significant differences ($p < 0.05$).

Abbreviation. WBC, white blood cell count; NEU, neutrophil count; Hb, hemoglobin; PLT, platelet count; ALB, albumin; CRE creatinine; TBIL, total bilirubin; IL-6, interleukin 6; CRP, C-reactive protein; P-SEP, presepsin; TIBC, total iron-binding capacity; UIBC, unsaturated iron-binding capacity; TSAT, transferrin saturation; FER, ferritin; HEP, hepcidin; LCN2, lipocalin-2.

Table S3

Correlations between items measured

	WBC	NEU	Hb	PLT	ALB	CRE	TBIL	IL-6	CRP	P-SEP	Iron	UIBC	TIBC	TSAT	FER	HEP	LCN2
WBC	1	0.96*	0.05	−0.10	−0.26	0.24	0.05	0.31*	0.47*	0.35*	−0.48*	−0.04	−0.26	−0.44*	0.00	0.30*	0.51**
NEU	0.96**	1	0.02	−0.16	−0.27	0.29	0.14	0.42*	0.55**	0.42*	−0.55**	−0.03	−0.28	−0.50**	0.03	0.39*	0.59**
Hb	0.05	0.02	1	0.02	0.52**	−0.24	0.30*	0.14	−0.01	−0.12	0.16	0.30*	0.36*	0.06	0.07	0.12	0.06
PLT	−0.10	−0.16	0.02	1	0.10	−0.35*	−0.49**	−0.39*	−0.36*	−0.42	0.27	0.09	0.18	0.25	−0.17	−0.33*	−0.20
ALB	−0.26	−0.27	0.52**	0.10	1	−0.19	0.17	−0.14	−0.30*	−0.40*	0.39*	0.63**	0.77**	0.14	−0.1	−0.04	−0.26
CRE	0.24	0.29	−0.24	−0.35*	−0.19	1	0.14	0.24	0.31*	0.44*	−0.27	−0.22	−0.31*	−0.22	0.05	0.31*	0.38*
TBIL	0.05	0.14	0.30*	−0.49*	0.17	0.14	1	0.29	0.17	0.30*	−0.07	0.12	0.11	−0.14	0.25	0.26	0.04
IL-6	0.31*	0.42*	0.14	−0.39*	−0.14	0.24	0.29	1	0.52**	0.41*	−0.55**	0.04	−0.20	−0.52**	0.05	0.60**	0.58**
CRP	0.47*	0.55**	−0.01	−0.36*	−0.30*	0.31*	0.17	0.52**	1	0.51**	−0.69**	−0.21	−0.47*	−0.58**	0.33*	0.60**	0.59**
P-SEP	0.35*	0.42*	−0.12	−0.42*	−0.40*	0.44*	0.30*	0.41*	0.51**	1	−0.45*	−0.23	−0.42*	−0.35*	0.23	0.33*	0.43*
Iron	−0.48*	−0.55**	0.16	0.27	0.39*	−0.27	−0.07	−0.55**	−0.69**	−0.45*	1	0	0.39*	0.93**	−0.04	−0.40*	−0.52**
UIBC	−0.04	−0.03	0.30*	0.09	0.63**	−0.22	0.12	0.04	−0.21	−0.23	0	1	0.90	−0.33*	−0.42*	−0.22	−0.06
TIBC	−0.26	−0.28	0.36*	0.18	0.77**	−0.31*	0.11	−0.20	−0.47*	−0.42*	0.39*	0.90**	1	0.06	−0.35*	−0.32*	−0.27
TSAT	−0.44*	−0.50**	0.06	0.25	0.14	−0.22	−0.14	−0.52**	−0.58**	−0.35*	0.93**	−0.33*	0.06	1	0.11	−0.32*	−0.48*
FER	0.00	0.03	0.07	−0.17	−0.10	0.05	0.25	0.05	0.33*	0.23	−0.04	−0.42*	−0.35*	0.11	1	0.52**	0.01
HEP	0.30*	0.39*	0.12	−0.33*	−0.04	0.31*	0.26	0.60**	0.60**	0.33*	−0.40*	−0.22	−0.32*	−0.32*	0.52**	1	0.38*
LCN2	0.51**	0.59**	0.06	−0.20	−0.26	0.38*	0.04	0.58**	0.59**	0.43*	−0.52**	−0.06	−0.27	−0.48*	0.01	0.38*	1

Note. Correlation coefficients with absolute values between 0.3 and 0.5 are marked * and those greater than 0.5 are marked **.

Abbreviation. WBC, white blood cell count; NEU, neutrophil count; Hb, hemoglobin; PLT, platelet count; ALB, albumin; CRE creatinine; TBIL, total bilirubin; CRP, C-reactive protein; P-SEP, presepsin; IL-6, interleukin 6; TIBC, total iron-binding capacity; UIBC, unsaturated iron-binding capacity; TSAT, transferrin saturation; FER, ferritin; HEP, hepcidin; LCN2, lipocalin-2.

Table S4

The eigenvectors of principal components 1 to 4

Analyte	PC1	PC2	PC3	PC4
WBC	0.35	0.13	-0.12	0.40
NEU	0.37	0.14	-0.10	0.36
Hb	−0.07	0.31	0.31	0.29
PLT	−0.15	−0.03	-0.21	0.45
ALB	−0.27	0.36	0.25	0.11
CRE	0.15	−0.02	-0.09	-0.09
TBIL	0.12	0.08	0.46	-0.20
IL-6	0.26	0.03	0.05	-0.36
CRP	0.03	0.02	0.17	0.01
P-SEP	0.37	0.10	0.28	-0.26
Iron	−0.30	−0.24	0.28	0.26
UIBC	−0.18	0.54	-0.09	-0.09
TIBC	−0.31	0.42	0.04	0.03
TSAT	−0.19	−0.42	0.22	0.22
FER	0.07	−0.09	0.25	0.15
HEP	0.26	0.07	-0.07	0.07
LCN2	0.27	0.12	0.49	0.14

Abbreviations. WBC, white blood cell count; NEU, neutrophil count; Hb, hemoglobin; PLT, platelet count; ALB, albumin; CRE creatinine; TBIL, total bilirubin; IL-6, interleukin 6; CRP, C-reactive protein; P-SEP, presepsin; TIBC, total iron-binding capacity; UIBC, unsaturated iron-binding capacity; TSAT, transferrin saturation; FER, ferritin; HEP, hepcidin; LCN2, lipocalin-2
